# Prospective Risk Factor Analysis for the Development of Post-operative Urinary Retention Following Ambulatory General Surgery

**DOI:** 10.1007/s00268-018-4697-4

**Published:** 2018-06-14

**Authors:** A. J. Scott, S. E. Mason, A. J. Langdon, B. Patel, E. Mayer, K. Moorthy, S. Purkayastha

**Affiliations:** 1 St Mary’s Hospital, Imperial College Healthcare NHS Trust, 10th Floor QEQM, London, W2 1NY UK; 2 Faculty of Medicine, Imperial College London, London, UK; 3 Prince of Wales Hospital, Sydney, Australia; 4 Department of Otolaryngology, Northwick Park Hospital, London, UK

## Abstract

**Aims:**

Post-operative urinary retention (POUR) is a common cause of unplanned admission following day-case surgery and has negative effects on both patient and surgical institution. We aimed to prospectively evaluate potential risk factors for the development of POUR following day-case general surgical procedures.

**Methods:**

Over a 24-week period, consecutive adult patients undergoing elective day-case general surgery at a single institution were prospectively recruited. Data regarding urinary symptoms, comorbidities, drug history, surgery and perioperative anaesthetic drug use were collected. The primary outcome was the incidence of POUR, defined as an impairment of bladder voiding requiring either urethral catheterisation, unplanned overnight admission or both. Potential risk factors for the development of POUR were analysed by logistic regression.

**Results:**

A total of 458 patients met the inclusion criteria during the study period, and data were collected on 382 (83%) patients (74.3% male). Sixteen patients (4.2%) experienced POUR. Unadjusted analysis demonstrated three significant risk factors for the development of POUR: age ≥ 56 years (OR 7.77 [2.18–27.78], *p *= 0.002), laparoscopic surgery (OR 3.37 [1.03–12.10], *p *= 0.044) and glycopyrrolate administration (OR 5.56 [2.00–15.46], *p *= 0.001). Male sex and lower urinary tract symptoms were not significant factors. Multivariate analysis combining type of surgery, age and glycopyrrolate use revealed that only age ≥ 56 years (OR 8.14 [2.18–30.32], *p *= 0.0018) and glycopyrrolate administration (OR 3.48 [1.08–11.24], *p *= 0.0370) were independently associated with POUR.

**Conclusions:**

Patients aged at least 56 years and/or requiring glycopyrrolate—often administered during laparoscopic procedures—are at increased risk of POUR following ambulatory general surgery.

**Electronic supplementary material:**

The online version of this article (10.1007/s00268-018-4697-4) contains supplementary material, which is available to authorized users.

## Introduction

An increasing proportion of general surgical procedures are performed as day cases, even amongst the elderly. Post-operative urinary retention (POUR) is defined as the inability to initiate micturition despite bladder distension in the early post-operative period [1]. POUR has a negative impact for both the patient and the hospital. Acute urinary retention can impair renal glomerular and tubular function [2]. POUR is frequently managed with urethral catheterisation which, in addition to being uncomfortable, carries risks of urinary tract infection, bleeding and trauma to the urogenital tract [3]. Additionally, many patients will require unplanned overnight admission which has direct cost implications for the healthcare organisation and exacerbates pressure on the availability of beds for emergency and elective admissions. POUR is believed to account for between 20 and 25% of unplanned inpatient admissions following day-case surgery [4, 5]. Alternatively, other patients may be discharged with a catheter *in situ,* requiring ongoing outpatient management in specialist clinics.

The reported incidence of POUR is highly variable (~ 2–50% [1]) and depends upon numerous procedural and patient-related factors. We recently published a meta-analysis of patient-related risk factors for the development of POUR following ambulatory general surgery [6]. Increased age (OR 2.11, 95% CI 1.15–3.86) and the presence of lower urinary tract symptoms (OR 2.83, 95% CI 1.57–5.08) increased the risk of POUR, while pre-operative alpha-blocker use was protective (OR 0.37, 95% CI 0.15–0.91). However, few of the available studies were prospective, contemporary and specifically considered POUR as a primary outcome. Consequently, we have limited reliable data regarding both the incidence of POUR following common ambulatory general surgical procedures and the risk factors associated with its development in the modern era. In this prospective observational cohort study, we aimed to address these deficiencies.

## Method

### Patients

A prospective observational study was conducted at St. Mary’s Hospital, London, UK, from May to October 2014 (24 weeks) with approval from the local research ethics committee (REC 14/SC/0219). Consecutive adult patients (age ≥ 18 years) undergoing elective day-case general surgical procedures (expected same-day discharge) were included. Patients undergoing bariatric, emergency or minor dermatological procedures and those with a urinary catheter in situ prior to, or inserted during, the procedure were excluded. Patients undergoing a procedure typically performed as a day case but with a planned overnight admission (e.g. because of attendant comorbidities) were not excluded.

### Data collection and analysis

Data regarding comorbidities, drug history, surgery and perioperative anaesthetic drug use were collected from the patient and their medical notes using a standardised, structured proforma (supplement). The International Prostate Symptom Score (IPSS) questionnaire [7] (supplement) was administered pre-operatively to patients who gave consent. Lower urinary tract symptoms (LUTS) were defined as either the presence of benign prostatic hyperplasia (BPH) or an IPSS ≥ 8. The total dose of opiate medication administered was calculated as milligrams of morphine equivalent.

The primary outcome was the incidence of POUR. Patients were defined as having POUR if two criteria were met: firstly, an impairment of bladder voiding within 24 h of surgery with a bladder volume of at least 500 ml on ultrasonic assessment; secondly, resultant urethral catheterisation and/or unplanned overnight admission. The majority of patients were catheterised if in painful retention but a minority of patients who were unable to void were admitted overnight for ongoing observation and were eventually able to void spontaneously (and so avoided catheterisation). This was at the discretion of the surgical team. Local emergency department attendances within 24 h of the primary surgical intervention were monitored for all patients to include those initially discharged who subsequently presented with urinary retention.

Data analysis was performed in R v3.3.1 (Foundation for Statistical Computing, Vienna, Austria) using the packages ‘epiR’, ‘pastecs’ and ‘OptimalCutpoints’. Association between potential risk factors and the development of POUR was assessed by bi- and multivariate logistic regression. Factors were selected on the basis of biological plausibility, scientific rationale, incidence and a low rate of missing data. The ability of regression models to discriminate between cases and controls was quantified using the area under the receiver operating characteristic curve (AUROC). Data are reported as mean (SD) for continuous data, as number (%) for binary data or as odds ratios with 95% confidence intervals. Significance was attributed at the 5% level.

## Results

**Fig. 1:**
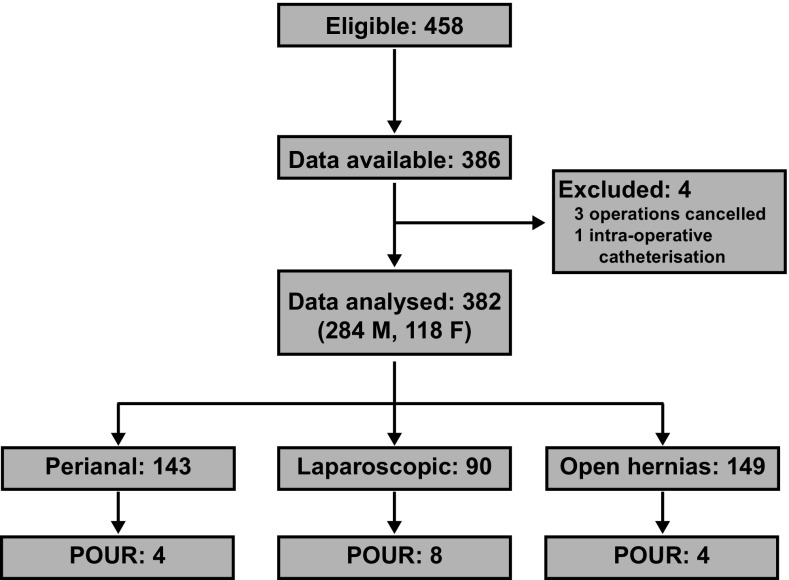
Patient recruitment, inclusion and primary outcomes

Figure 1 details patient recruitment and outcome data. A total of 458 patients met the inclusion criteria during the study period, and data were collected on 386 (84%) patients. This was due to a lack of available research personnel to collect data at every operating list. A total of 382 patients (284 [74.3%] male) were subsequently analysed, with four excluded due to cancellation of surgery (*n* = 3) or intra-operative catheterisation (*n* = 1). Operations were categorised into three groups: perianal procedures (e.g. examination of the anorectum under anaesthesia and treatment of fistulas, fissure-in-ano, haemorrhoidal disease and warts), laparoscopic procedures (e.g. laparoscopic hernia repair, cholecystectomy and fundoplication) and open hernia repair (e.g. inguinal and ventral hernioplasty). The overall incidence of POUR was 16/382 (4.2%) with 12 patients requiring a catheter (mean age 58 years) and four patients ultimately not requiring catheterisation but forced to stay overnight while waiting to micturate (mean age 63 years). Eight of the catheterised patients had an unplanned overnight admission. The incidences of POUR were 2.8, 9.8 and 2.9% in patients undergoing open hernia repair, laparoscopic procedures and perianal procedures, respectively.

Table 1 describes the unadjusted association between the incidence of POUR and potential risk factors. Increasing age (OR 1.04, 95% CI 1.00–1.07, *p *= 0.018), laparoscopic surgery (OR 3.37, 95% CI 1.03–12.10, *p *= 0.044) and glycopyrrolate administration (OR 5.56, 95% CI 2.00–15.46, *p *= 0.001) demonstrated a significant association with the development of POUR. AUROC analysis demonstrated that the optimal cut-off for age was 56 years which was associated with an OR of 7.77 (95% CI 2.18–27.78, *p *= 0.002) for the development of POUR. Neither male sex (OR 0.79, 95% CI 0.28–2.23, *p *= 0.661) nor the presence of LUTS (OR 0.58, 95% CI 0.13–2.64, *p *= 0.488) were significant risk factors.

**Table 1:** Bivariate analysis by logistic regression of potential risk factors for the development of POUR

Factor	POUR	No POUR	OR	95% CI	*p* value
Total (*n* = 382)	16	366			
Sex (*n* = 382)					
Female (*n* = 124)	6	118	Ref		
Male (*n* = 258)	10	248	0.79	0.28–2.23	0.661
Age (years, *n* = 382)	59.5	49.1	1.04	1.00–1.07	0.018
Age < 56 years (*n* = 238)	3	235	Ref		
Age ≥ 56 years (*n* = 144)	13	131	7.77	2.18–27.78	0.002
IPSS (*n* = 155)					
IPSS < 8 (*n* = 97)	3	94	Ref		
IPSS ≥ 8 (*n* = 58)	2	56	1.12	0.18–6.90	0.904
BPH (*n* = 244)^a^					
No BPH (*n* = 224)	9	215	Ref		
BPH (*n* = 20)	1	19	1.26	0.15–10.46	0.832
LUTS (*n* = 379)					
No LUTS (*n* = 306)	14	292	Ref		
LUTS (*n* = 73)	2	71	0.58	0.13–2.64	0.488
Surgery type (*n* = 382)					
Open hernia (*n* = 149)	4	145	Ref		
Laparoscopic (*n* = 90)	8	82	3.37	1.03–12.10	0.044
Perianal (*n* = 143)	4	139	0.84	0.26–4.25	0.953
Operative time (min, *n* = 373)	43.4	39.8	1.00	0.98–1.01	0.644
Morphine dose (mg, *n* = 357)	20.6	19.4	1.00	0.98–2.03	0.760
Glycopyrrolate use (*n* = 357)					
Glycopyrrolate not used (*n* = 297)	8	289	Ref		
Glycopyrrolate used (*n* = 60)	8	52	5.56	2.00–15.46	0.001
Age ≥ 56 years and glycopyrrolate (*n* = 357)					
Age < 56 years or glycopyrrolate not used (325)	9	316	Ref		
Age ≥ 56 years and glycopyrrolate used (*n* = 32)	7	25	9.83	3.38–28.61	<0.001

Number of patients available for analysis indicated in brackets. Number of patients or mean value is given for each outcome for categorical and continuous variables (e.g. age) respectively. LUTS was defined as an IPSS ≥ 8 and/or BPH

^a^BPH could only be recorded in male patients (*n* = 258), and data were not available for 14 patients

We constructed a multivariate model combining type of surgery, age ≥ 56 years and glycopyrrolate administration. Only age (OR 8.14, 95% CI 2.18–30.32, *p *= 0.0018) and glycopyrrolate use (OR 3.48, 95% CI 1.08–11.24, *p *= 0.0370) were independently associated with POUR (Table 2). Although patients undergoing laparoscopic surgery demonstrated a trend towards an increased risk of POUR in this model (OR 3.76, 95% CI 1.00–14.16, *p *= 0.0507), a model combining type of surgery with glycopyrrolate use confirmed that laparoscopy was not an independent risk factor per se (OR 2.30, 95% CI 0.63–8.42, *p *= 0.2084). A model including glycopyrrolate use and age ≥ 56 years had an AUROC of 0.79 (95% CI 0.67–0.90). Patients ≥ 56 years who also received glycopyrrolate had an odds ratio of 9.83 (95% CI 3.38–28.61, *p *< 0.001) on bivariate analysis for the development of POUR compared to younger patients or those not receiving the anticholinergic (Table 1).

**Table 2:** Multivariate logistic regression models of potential risk factors for the development of POUR

Model	POUR	No POUR	OR	95% CI	*p* value
Glycopyrrolate + age ≥ 56 years + surgery type					
Total (*n* = 357)	16	341			
Glycopyrrolate used			3.48	1.08–11.24	0.0370
Age ≥ 56 years			8.14	2.18–30.32	0.0018
Perianal surgery			2.27	0.51–10.04	0.2790
Laparoscopic surgery			3.76	1.00–14.16	0.0507
Glycopyrrolate + surgery type					
Total (*n* = 357)	16	341			
Glycopyrrolate			4.48	1.37–14.67	0.0132
Perianal surgery			1.48	0.34–6.39	0.5964
Laparoscopic surgery			2.30	0.63–8.42	0.2084
Glycopyrrolate + age ≥ 56 years					
Total (*n* = 357)	16	341			
Glycopyrrolate used			4.41	1.54–12.62	0.0056
Age ≥ 56 years			6.32	1.74–23.00	0.0051

## Discussion

With a shrinking bed base and a rising number of ambulatory general surgical procedures, it is important to quantify the incidence of POUR, a common cause of readmission after surgery, and understand its contributory risk factors [4, 5]. However, there are currently deficiencies in the literature as many studies were published over a decade ago, are retrospective, do not consider POUR as a primary outcome and do not specifically concern patients undergoing day-case general surgical procedures. The present study design is therefore novel in the context of the literature and robustly demonstrates that while POUR is not common overall following day-case general surgery, patients over 56 years and those receiving glycopyrrolate are at significantly increased risk.

The high variation in the quoted incidence of POUR within the literature reflects the lack of a standardised definition in addition to procedure- and patient-specific differences. The overall incidence of POUR in this prospective investigation was 4.2%. Given the aforementioned dearth of prospective studies looking at day-case general surgical procedures, it is difficult to find accurate figures for direct comparison within the literature. However, a prospective investigation assessing POUR following a variety of surgical procedures found an overall incidence of 4.8% [8] and a large (400,000 patients) retrospective cohort study reported an incidence of 2.1% in a similar cohort [9]. Therefore, our figure seems reasonable.

Increasing age, glycopyrrolate administration and laparoscopic surgery were associated with the development of POUR on bivariate analysis, but only age and glycopyrrolate use remained significant on multivariate logistic regression. Other studies have identified age as independent risk factor for POUR following day-case surgery and our recent meta-analysis suggested a high-risk cut-off at 60 years [6]. In the present study, ROC analysis yielded a cut-off of 56 years, at or above which the incidence of POUR was 9% compared to 1% below 56 years (relative risk 7.1). The precise reason why older patients are at higher risk of POUR is not entirely understood, though other authors have speculated that degradation of relevant spinal pathways may be responsible [10].

Reversal of neuromuscular blockade with acetylcholinesterase inhibitors (such as neostigmine) is associated with significant unwanted muscarinic side effects (e.g. bradycardia). Therefore, such reversal agents are co-administered with glycopyrrolate, a muscarinic receptor antagonist. Bladder detrusor contraction and internal urethral sphincter relaxation during micturition are controlled by parasympathetic stimulation via muscarinic receptors; therefore, blockade of these receptors by glycopyrrolate may induce urinary retention. Indeed, we found that administration of glycopyrrolate was significantly associated with the development of POUR (relative risk 5.0). Muscle relaxation is typically required for laparoscopic procedures, and many patients will require reversal, depending on the degree of residual paralysis at the end of the procedure. It is therefore logical that the apparent association between laparoscopic surgery and POUR on bivariate analysis was actually confounded by glycopyrrolate administration. Atropine is another antimuscarinic agent, and Dreijer et al. demonstrated an increased risk of POUR (OR 5.9) in association with its use in their prospective analysis [8]. Atropine was only administered to one patient in our study, so analysis of its effect could not be performed.

Many factors have been associated with POUR in other investigations including procedure duration, intra-operative fluid administration, regional anaesthesia, sex, diabetes, LUTS and opiate administration. We did not replicate these findings in the present study though heterogeneity between study populations makes comparisons difficult. Furthermore, many others were based on patient cohorts undergoing longer, more physiologically significant interventions. Procedure duration, fluid administration, opiate use and anaesthesia type were all fairly uniform in this cohort undergoing day-case surgery, and so, it is perhaps unsurprising that significant differences were not noted. In particular, we found no association between male sex or LUTS and POUR. The lack of association with male sex is supported by our previous meta-analysis [6] and findings in other surgical disciplines [8, 11]. LUTS is poorly defined in the literature, so we used a validated (in both males [7] and females [12, 13]), objective questionnaire to determine symptomatic voiding dysfunction and defined LUTS as either an IPSS ≥ 8 and/or known BPH. In contrast to the results of our previous meta-analysis, we did not demonstrate any association between the presence of LUTS and POUR. It is difficult to draw firm conclusions in this regard as, due to logistical constraints, we were only able to collect completed IPSS questionnaires from approximately 40% of our cohort, of which only five experienced POUR. This limits assessment of the use of IPSS in predicting POUR though it is noteworthy that another study also concluded that IPSS was not useful in this regard [14].

We consider this investigation to have several strengths. Its prospective nature eliminates sources of bias associated with retrospective studies and allows incidence to be accurately calculated. Defining POUR simply as an inability to micturate with bladder distension would be to ignore the fact that for many patients this is temporary and can resolve rapidly with conservative measures (e.g. ambulation and the sound of running water). Our definition of POUR required a patient to be catheterised or admitted overnight due to an inability to micturate, reflecting retention that failed to respond rapidly to conservative management. This definition is of direct relevance to both hospital and patient and is more conservative than simply measuring bladder volumes post-operatively which can over-diagnose POUR—a study of routine post-operative bladder ultrasound found that nearly 50% of patients had volumes > 500 ml but only half of these patients required catheterisation due to voiding failure [11]. Our patient population was a well-defined, extremely generalisable cohort undergoing routine general surgical procedures performed widely.

However, we also acknowledge several limitations in our study. Firstly, data related to anaesthesia were collected from patient charts and therefore subject to error if drug administration was not accurately recorded. In particular, we were unable to analyse fluid administration in a meaningful way as most patients simply received a one-litre bag of crystalloid but the volume actually infused was unrecorded. Secondly, the total number of patients experiencing POUR in our investigation was relatively low (16 of 382); a larger patient cohort would improve the statistical models. In particular, the estimation of effect sizes would be more accurate, but it is unclear whether other risk factors for POUR would have been demonstrated as no other factors even showed a trend towards significance on bivariate modelling. Lastly, we could not meaningfully analyse some other potential risk factors, such as the presence of diabetes or spinal/epidural anaesthesia, because the incidence of cases was too low. However, the effects of spinal/epidural anaesthetic on bladder function are well documented and this modality is generally avoided in day-case procedures for precisely this reason [1].

In this prospective investigation, we have found that glycopyrrolate administration and/or age ≥ 56 years significantly increases the risk of POUR following day-case general surgery. Glycopyrrolate is commonly administered during laparoscopic surgery and appears to explain the increased risk of POUR seen following these procedures compared to perianal and open hernia surgery. The incidence of POUR in patients aged over ≥ 56 years and receiving glycopyrrolate was 22% (relative risk 7.9). With an ageing population and increasing use of laparoscopy (frequently requiring muscle relaxation), it is important to be aware that this cohort has a high risk of POUR and to counsel patients and plan operative lists accordingly. Alternatively, it may be possible to avoid using glycopyrrolate in high-risk patients as there are options other than neostigmine and glycopyrrolate for reversing muscle paralysis. For example, the drug sugammadex encapsulates paralytic agents, preventing blockade of the neuromuscular junction without muscarinic side effects and therefore does not require co-administration with an antimuscarinic. Sugammadex is more expensive than neostigmine/glycopyrrolate and can only be used to reverse vecuronium or rocuronium, but economic analysis has suggested that it may be cost-effective due to the value of time saved by more rapid reversal of paralysis [15]. A reduction in the incidence of POUR with reversal by sugammadex compared to neostigmine/glycopyrrolate administration has the potential for further cost reductions but would need prospective investigation.

## Electronic supplementary material

Below is the link to the electronic supplementary material.

Supplementary material 1 (PDF 104 kb)
