# Supplementary material for: Prospective Risk Factor Analysis for the Development of Post-operative Urinary Retention Following Ambulatory General Surgery
Source: World J Surg. 2018 Jun 14;42(12):3874–9. doi: 10.1007/s00268-018-4697-4 (PMC6244976; doi:10.1007/s00268-018-4697-4)
Supplement: Supplementary file 1 — Supplementary material 1 (PDF 104 kb) [file 268_2018_4697_MOESM1_ESM.pdf]

Prospective Risk Factor Analysis for the Development of Post-Operative Urinary Retention Following Ambulatory General Surgery

Alasdair J Scott PhD[†], Sam E Mason MBBS[∂†], Angharad J Langdon MBBS[‡], Bhavesh Patel MBBS[§], Erik K Mayer PhD[*†], Krishna Moorthy MD[*†] and Sanjay Purkayastha MD[*†]*

[*] St. Mary's Hospital, Imperial College Healthcare NHS Trust, London, UK

[†] Department of Surgery & Cancer, Imperial College London, UK

[∂] North Middlesex University Hospital, London, UK

[‡] Prince of Wales Hospital, Sydney, Australia

[§] Department of Otolaryngology, Northwick Park Hospital, London

Supplementary Material

Data Collection Proforma

Patient Questionnaire

Imperial College Healthcare NHS
NHS Trust

Data Collection Proforma V1

Patient ID ☐

Demographic Data

**Sticker / Pt Details**	**Procedure**	**Op Date**
Name<br>DOB<br>MRN	**Planned Overnight Stay?**<br>Yes ☐ No ☐	

Pre-Operative Data

Co-morbidities *(please state diagnosis)*

☐ **None**

Diabetes: Type 1 ☐ or Type 2 ☐

Prostate: BPH ☐ or Ca ☐

☐ Previous pelvic surgery / radiotherapy:

…………………………………………………….

☐ Other significant: e.g. neurological

……………………………………………………

Pre-operative medications

☐ **None**

**α-blocker**	**5α-reductase inhibitor**
☐ Tamsulosin<br>☐ Doxazosin<br>☐ Prazosin	☐ Finasteride<br>☐ Dutasteride
**Anti-cholinergic**	**Pro-cholinergic**
☐ Oxybutynin<br>☐ Tolterodine<br>☐ Solifenacin<br>☐ Fesoteradine	☐ Donepazil<br>☐ Rivastigmine<br>☐ Galantamine

Other Significant:……………………………………………………

Patient Questionnaire: ☐ Not Done

☐ Previous bladder catheterisation

☐ Other condition affecting urination:

……………………………………………………

**Total IPSS Score**	**Voiding Score** *Qs. A, C, E and F*	**Storage Score** *Qs. B, D and G*
☐	☐	☐

Peri-Operative Data

Total IVI Volume (ml)

Intra-operative catheterisation?
Yes ☐ No ☐

Anaesthetic Route: *may be more than one*

☐ General
☐ Spinal
☐ Epidural
☐ Local Block
(not including infiltration at end of op)

Peri-operative medications: *pre-med, intra-op and recovery*

**Anti-cholinergic**	**Sympathomimetic**
☐ Glycopyrrolate<br>☐ Atropine<br>☐ Hyoscine	☐ Metaraminol<br>☐ Ephedrine<br>☐ Phenylephrine

Opiates (dose)

☐ Morphine…………………	☐ Remifentanyl (IVI)
☐ Fentanyl…………………	☐ Tramadol…………………
☐ Alfentanyl………………..	☐ Codeine…………………..

Outcome

Post-operative urinary retention? **No** ☐ **Yes** ☐ **Residual (ml)** ☐
Pt. required catheterisation due to inability to micturate within 24hrs of procedure.

Did POUR result in an unplanned overnight stay? **No** ☐ **Yes** ☐

If not in POUR, did patient stay overnight because of delay in passing urine? **No** ☐ **Yes** ☐

**Date of discharge**	**Any other comments**

Post-Operative Urinary Retention Score

Patient Questionnaire – Version 2, 04/03/2014

Imperial College Healthcare NHS
NHS Trust

Researcher Use Only

Total Score: ☐

1. Have you ever had a bladder catheter because of difficulty passing urine? **Yes – ☐** **No – ☐**

2. Do you regularly suffer from constipation? **Yes – ☐** **No – ☐**
– e.g. passing stool less than 3 times per week
– e.g. straining or passing hard stool more than a quarter of the time

3. If you have a condition which affects your ability to urinate or control urination, please note it below.

____

4. Urinary Function: please circle the best answer for each of the 7 questions below

	Not at all	Less than 1 time in 5	Less than half the time	About half the time	More than half the time	Almost always
**A Incomplete Emptying**<br>Over the past month, how often have you had the sensation of not emptying your bladder completely after you have finished urinating?	0	1	2	3	4	5
**B Frequency**<br>Over the past month, how often have you had to urinate again less than two hours after you last finished urinating?	0	1	2	3	4	5
**C Intermittency**<br>Over the past month, how often have you found you stopped and started again several times when you urinated?	0	1	2	3	4	5
**D Urgency**<br>Over the past month, how often have you found it difficult to postpone urination?	0	1	2	3	4	5
**E Weak Stream**<br>Over the past month, how often have you had a weak urinary stream?	0	1	2	3	4	5
**F Straining**<br>Over the past month, how often have you had to push or strain to begin urination?	0	1	2	3	4	5

	None	Once	Twice	3 times	4 times	5 or more times
**G Nocturia**<br>Over the past month, how many times did you usually get up each night to urinate from the time you went to bed until the time you got up in the morning?	0	1	2	3	4	5
